# Feasibility of Ecological Momentary Assessment in the Indian Context to Address Challenges Associated with Hearing-Aid Use

**DOI:** 10.1055/s-0045-1811655

**Published:** 2026-03-03

**Authors:** Puttaraju Sahana, Chaithra M. Chandrashekhar

**Affiliations:** 1 Center of Excellence (C-PEC), All India Institute of Speech and Hearing (AIISH), Mysore, Karnataka, India; 2 Department of Audiology, All India Institute of Speech and Hearing (AIISH), Mysore, Karnataka, India

**Keywords:** hearing aid outcomes, momentary analysis, hearing impairment, validity

## Abstract

**Introduction:**

Ecological momentary assessment (EMA) involves the repeated real-time or near real-time sampling of individuals' behaviors and experiences within their natural settings, offering insights into the daily challenges experienced by individuals with hearing impairment.

**Objective:**

The study aims to evaluate EMA's feasibility and construct validity in the Indian context, assessing its effectiveness in analyzing hearing aid outcomes at different times.

**Methods:**

Twelve participants, aged between 49 and 66 (mean: 57.83) years, with bilateral hearing loss, participated in the study. A prospective cohort study was conducted from July to November 2023. Ecological momentary assessments were administered using a questionnaire hosted on Microsoft Forms (Microsoft Corp.), with links sent via WhatsApp (Meta Platforms, Inc.) 3 times daily over 10 days. On average, participants completed 3 surveys daily, each consisting of 15 questions.

**Results:**

A total of 237 entries was collected, with a 65.83% compliance rate. Most entries highlighted the evident advantages of hearing aids, especially in quiet surroundings. Utilizing Friedman's and Wilcoxon signed-rank tests for comparative analysis, significant variations were observed in EMA entries over time within identical listening environments.

**Conclusion:**

The benefits of hearing aids in quiet environments were emphasized due to their capacity to restore audibility. The significant variations in individual preferences within the same environment emphasize the necessity for personalized hearing aid settings. These discrepancies in EMA entries highlight the importance of incorporating objective background noise measurements in surveys to optimize hearing aid performance. Ecological momentary assessment surveys provide valuable insights into listener preferences, facilitating optimal adjustments.

## Introduction

Audiologists in India mostly rely on self-report questionnaires to gain insights into the hearing difficulties experienced by adults with hearing impairment (HI). Standardized self-report questionnaires are commonly utilized to assess the impact of HI on an individual's activity, participation, and quality of life. 1 The response to such questionnaires depends on the individual's memory of different listening environments and recall abilities. 2 3 Thus, recall bias is inherent in retrospective self-report questionnaires. 4

The field of audiology has embraced the adoption of ecological momentary assessment (EMA) to gain insights into the everyday challenges faced by individuals with HI. It involves repeated sampling of individuals' behaviors and experiences in real-time or near real-time, taking place within their natural settings. This innovative approach enables individuals to provide real-time descriptions of their experiences within their natural environments. Ecological momentary assessment can be a promising tool in providing a comprehensive understanding of the hearing difficulties experienced by individuals with HI in everyday situations. 1

The data collected through EMA can provide valuable insights into the fluctuations, patterns, and contexts of an individual's experiences and behaviors. It allows researchers to capture moment-to-moment variability, understand the impact of situational factors on psychological states, and examine how different aspects relate to one another over time. Participants are typically prompted at various times throughout the day to report on their experiences, thoughts, emotions, behaviors, or other variables of interest. These prompts can be triggered randomly, at predetermined intervals, or in response to specific events or activities.

In individuals fitted with hearing aids (HAs), EMA can be beneficial in providing information on the effectiveness of HA in different listening environments. Overall, EMA provides a powerful and flexible approach to studying individuals' experiences and behaviors in their natural environments, contributing to a more pronounced understanding of human psychology and behavior.

The efficacy of HAs is commonly assessed in a controlled laboratory environment, but these outcomes may not accurately reflect real-world scenarios. Therefore, a momentary analysis of an individual's listening situations can offer a more comprehensive understanding of the participant's unique listening profile. Thus, the present study aims to assess the feasibility and construct validity of EMA in the Indian scenario, and its effectiveness in evaluating HA outcomes at various times.

## Methods

A prospective cohort study design was framed to evaluate the aim of the study. The study was conducted from July 2023 to November 2023, and the development and validation of the questionnaire took place in June 2023.

### Participants

All individuals using bilateral HAs who visited the Department of Audiology at the All India Institute of Speech and Hearing, Mysore, from July to November 2023 were informed about how the study worked and the necessity of providing 3 samples per day for a period of 10 days. Only those who consented to participate and were willing to meet this requirement were considered for the study. The inclusion criteria required bilateral hearing loss ranging from moderate to moderately severe, a minimum of six months of bilateral HA use, and unaided speech perception scores exceeding 60%. Individuals who met these criteria were subsequently included in the study.

Twelve participants in the age range of 49 to 66 years (mean age: 57.83 years) were recruited from the Department of Audiology at the All India Institute of Speech and Hearing in Mysore. The demographic details of the participants can be seen in Table 1 .

**Table 1:** Demographic details of the participants in the study

		N
Age	< 55 years	5
	56–65 years	7
Employment status	Employed	6
	Unemployed	6
Hearing aid type	BTE users	7
	RIE users	5

**Abbreviations:** BTE, behind the ear; RIE, receiver in ear.

*Procedure:* The study protocol adhered to the institutional ethical guidelines for behavioral research on human subjects, 5 which is also in line with the Declaration of Helsinki. 6 Before conducting any experiment, the participants provided informed consent. The EMAs were carried out for a period of 10 days. The participants, on average, took 3 surveys per day, each containing 15 questions. The EMA procedure complied with the suggested guidelines for EMA study design followed by other health disciples. 7 8 9 10 11 12

The questionnaire was adapted from Danielle et al. 13 and was initially used for children. The original questionnaire was modified to suit our study population ( Appendix 1 ). Three audiologists, each with 3 to 5 years of clinical experience, validated the questions for their appropriateness and relevance to challenges associated with hearing difficulties. Based on their feedback, minor modifications were made to improve clarity and ensure better alignment with real-world hearing difficulties. Only the revised and validated questions were included in the final questionnaire. Subsequently, the questions were translated into Kannada. Two Kannada teachers performed the forward translation with an emphasis on the meaning conveyed. Following this, the translated Kannada questions were administered to five individuals with HI who were not part of the study. They provided binary responses (yes or no) on their understanding of the questions. Those identified as difficult to comprehend were then revised for clarity.

The questions were hosted in Microsoft Forms (Microsoft Corp.), and the link was sent via WhatsApp (Meta Platforms, Inc.) application 3 times a day (11.00, 15.00 & 19.00 IST). The prompts were given primarily through WhatsApp. However, a phone call was given if the response was not received within 15 minutes. Each participant was given a brief outline of the study methodology and was informed about how to withdraw from the study in case of any inconvenience. Participants who were comfortable using Android phones and could follow the instructions were considered for the study.

The survey consisted of two sets of questions. Set 1 focused on gathering responses related to the listening situation and environment. In contrast, set-2 questions were explicitly asked when the HA was not helpful. These questions aimed at clarifying the effectiveness of the HA and enquire about any additional support needed by the participant.

## Results

The EMA survey was scheduled thrice daily over 10 days, resulting in 30 surveys per participant. Throughout the trial, participants completed 237 EMA entries, demonstrating an average compliance rate of 65.83%. Compliance was determined by the ratio of completed surveys (237) to the target of 360 surveys (12 participants, 3 surveys per day per participant, over a 10-day data collection period). On average, participants submitted 19.5 entries per trial (standard deviation = 3.20, maximum = 29 & minimum = 16). Additionally, an analysis was conducted to assess the compliance rates among employed and unemployed participants separately. The findings revealed that out of a total of 237 entries, only 83 were from participants who were employed, constituting 23.05% of the sample.

### Reported Benefit from HAs

**Fig. 1:**
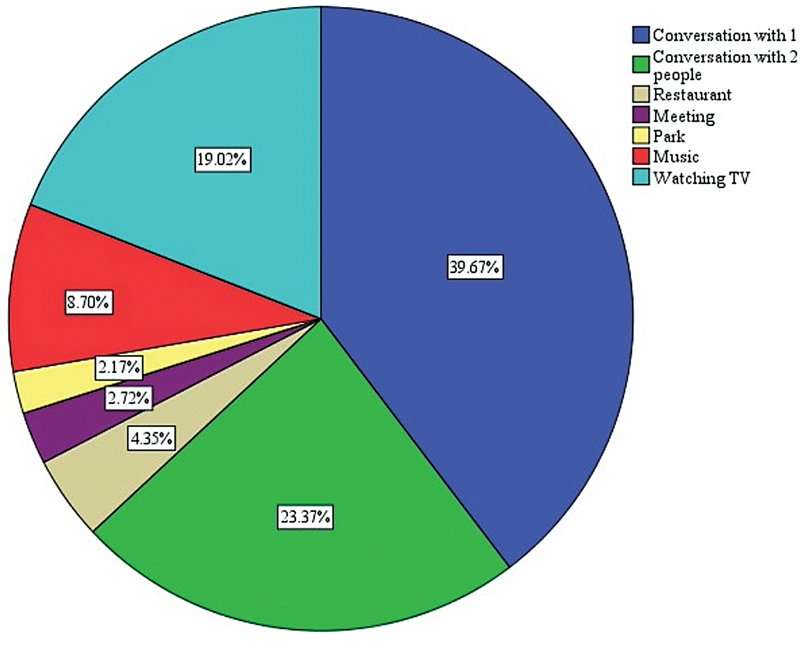
Pie chart depicting the percentage of various listening environments in which hearing aids have demonstrated their effectiveness.

The EMA entries were sorted into responses for the sets-1 and -2 questionnaires. Set-2 responses were only answered when the HA did not prove beneficial. There were 54 entries on set-2 and 183 entries on set-1. Almost 77% of EMA entries highlighted clear benefits of HAs. Further analysis was conducted based on the specific listening environments encountered by the participants. Descriptive statistics showed that the majority of participants experienced a quiet environment (64.4%), followed by slightly noisy (29.5%), moderately noisy (2.7%), and noisy (3.2%) listening environments. Fig. 1 illustrates the distribution of reported listening situations by the participants. Also, participants expressed that without the HAs in the current scenario, they would either feel helpless (82.15%) or they could adapt 15.85% of the time. Nevertheless, most EMA entries emphasized the absolute necessity of HAs in the listening environments.

### Limited Benefit from HAs

The set-2 questionnaire aimed to address the specific issues faced by participants when the HA provided limited benefit. Among the 54 entries, 35 (64.8%) indicated that participants felt embarrassed due to difficulty hearing in the current situation. Additionally, a minority of participants expressed feelings of frustration (24.04%), while 11.1% reported experiencing depression in such situations.

Among the reported hearing difficulties, a small percentage (5.5%) indicated challenges in localization, while 12.2% expressed dissatisfaction with the HA battery affecting audibility. A significant number of entries highlighted that the person's voice was perceived as somewhat soft (50%), and understanding speech required considerable effort (92%), resulting in only partial comprehension of the information (74.04%).

Most entries emphasized the need for more explicit speech (74.07%), enhanced noise cancellation (62.2%), and improved audibility (40%) from their HAs. To cope with these challenges, many participants resorted to compensatory mechanisms, such as relying on lip reading (53.7%), requesting repetitions (27.7%), and sometimes pretending to have understood everything (18.6%). Unfortunately, a substantial portion of entries (81.4%) expressed an intention to avoid similar listening environments in the future.

### Comparison among EMAs for the Same Participant

A Friedman's test was conducted to identify potential variations in EMA entries for the same participant when there was minimal HA benefit in the same listening environment. For example, the comparison was made for more than two EMA entries while the participant was watching television. The findings indicated a significant difference in EMA entries across different samples when conversing with 2 or more people (Chi-squared [χ ^2^ ] [8] = 25.39; *p* = 0.001).

Subsequently, the Wilcoxon signed-rank test was utilized to analyze pairs of samples. The results demonstrated that the 2 samples obtained exhibited differences in the context of meetings (Z = 1.19; *p* = 0.03) and restaurants (Z = 2.21; *p* = 0.04). However, no significant difference was observed for EMA entries while watching television (Z = 1.41; *p* = 1.57). This implies that, when faced with identical situations, participants sought various feelings, problems, and dissimilar adjustments to be implemented in their HAs, as evidenced by distinct EMA entries over time.

## Discussion

The study sought to investigate the practicality of utilizing EMAs in the Indian context and to evaluate its efficacy in examining challenges related to HA usage. The compliance rate was 65.83%, lower than the rates reported in previous studies. 1 14 Notably, there was a substantial variation in completing EMA entries among participants. The active participants completed all three surveys in a single day, whereas the passive participants did not submit any or completed only one EMA survey. A notable portion of the recorded EMA entries came from these active participants who are unemployed or retired. There are various reasons for the observed difference in compliance rates compared to prior research. One could be attributed to the employment status of many participants in our study, potentially impeding their prompt response to EMA surveys. Another factor might be that the questionnaire length could have led participants to disregard it in their work schedules. 15

In addition, nearly 77% of the EMA entries indicated a substantial benefit from HAs, with most participants reporting quiet listening environments. Among those entries, participants stated that they experienced a quiet environment approximately half the time each day. Most EMA entries were obtained from retired participants who spend significant time at home. Additionally, it is possible that participants found it more convenient to engage in the EMA task at home rather than in outdoor environments that necessitate active communication. 15 Analyzing the timing of EMA survey reception, it was promptly received in a home environment but frequently postponed in outdoor settings. Additionally, there may be instances in which a postponement is forgotten, resulting in the survey not being completed. In a quiet environment, enhancing audibility for individuals with HI can significantly improve performance. 16 Thus, HAs, by increasing audibility, contributed to improved performance. 17 18 19

In addressing the challenges associated with HA usage, the majority reported feeling embarrassed in situations in which they did not comprehend what was being said. This is on par with the previous reports. 20 21 A greater proportion of participants reported no localization challenges, likely because all participants wore bilateral HAs, which could aid in detecting the direction of sounds. 22 23 The EMA entries disclosed that HAs do not offer sufficient benefits in noisy environments. This holds true for many individuals with HI, emphasizing that restoring audibility may not guarantee effective speech understanding in challenging situations. 24 Most of the entries revealed that the speech requires more clarity, advanced noise cancellation and more audibility. The intricate effects of sensorineural hearing loss (SNHL) on speech understanding are influenced by multiple physiological factors. Conventionally, it is reported that elevated threshold, broader bandwidth, and temporal-processing deficits are the primary factors contributing to the speech understanding challenges faced by these individuals. 24 25 Therefore, restoring the audibility will not improve speech compression especially in background noise.

The EMA entries from the same participants in identical listening environments exhibited significantly diverse responses. One possible reason for this inconsistency could be the background noise level. For instance, when participants visited a restaurant, the background noise was significantly higher in the evening, requiring more emphasis on noise cancellation rather than audibility. This limitation highlights the need for an automatic objective measurement of background noise during the EMA survey. Such an objective measurement and momentary analysis could serve as a reliable indicator for audiologists to obtain a deeper understanding of optimizing HA features.

Nevertheless, subjective EMA provides real-world data based on the participant's listening environment. Future research can focus on developing an EMA app with background noise measurement. Additionally, EMA can be used to assess the effectiveness of two different features and types of HAs.

Furthermore, although EMA studies are one among the current research trends, there are limitations in the Indian context. Participants may not consistently adhere to the study protocol, leading to missing data, self-reporting bias can lead to incorrect data, technology constraints (compatibility, availability if internet) and privacy concerns arise as the data is being collected in real time. Future studies could explore strategies such as incentives or real-time monitoring to enhance compliance rate.

## Conclusion

Ecological momentary assessment serves as a tool to comprehend the participant's current listening environment and assess the effectiveness of the hearing aid in those situations. However, the low compliance rate of 65.83% indicates that conducting multiple surveys may not be feasible, especially for employed participants. The substantial variations in individual preferences within the same environment require the need for personalized fine tuning of HA settings. Furthermore, EMA survey provides insights into variations in the listener's preferences and helps with optimizing the fitting of the HA.
