# Supplementary material for: Feasibility of Ecological Momentary Assessment in the Indian Context to Address Challenges Associated with Hearing-Aid Use
Source: Int Arch Otorhinolaryngol. 2026 Mar 3;30(1):1–5. doi: 10.1055/s-0045-1811655 (PMC12956388; doi:10.1055/s-0045-1811655)
Supplement: Supplementary file 1 — Supplementary Material [file 10-1055-s-0045-1811655-s241819.pdf]

Appendix 1

This survey will help understand the hearing difficulties faced by you. Please take 5-10 mins to help us know you better

SET 01

1. What are you up to currently?
   a. Conversation with one
   b. Conversation with 2 or more
   c. Eating in a restaurant or just at a restaurant
   d. Attending a meeting
   e. Taking a walk, in park
   f. Listening to music
   g. Watching TV
2. Which word describes the room or space/environment you are in right now?
   a. Quiet
   b. Slight noisy
   c. Moderately Noisy
   d. Very Noisy
3. How often do you encounter such a listening situation?
   a. Very often
   b. Half of the time in a day
   c. Rarely
4. How do you think you'd have felt without HAs in this situation?
   a. Helpless
   b. Cannot manage the situation
   c. Able to adjust
   d. Can manage
5. Is the hearing aid helping you in the present situation?
   a. Absolutely
   b. Somewhat yes
   c. Not at all

If your answer for 5 is option B and/or C, Kindly fill in the next set of questions

SET 02

1. How do you feel the present situation with HA put on?
   a. Frustrated
   b. Embarrassed
   c. Depressed
2. Are you able to localize or determine the direction of sounds in your current environment?
   a. Yes, accurately
   b. Somewhat
   c. Not at all
3. Are you able to hear and understand background sounds or noises in your current environment?
   a. Yes, clearly
   b. Somewhat
   c. Not at all
4. How loud do people's voices sound right now?
   a. Too loud
   b. A bit loud
   c. Just right
   d. A bit soft
   e. Too soft

 © 2026. The Author(s).

5. How well do you understand speech right now?
   a. Almost everything
   b. Something
   c. Almost nothing
6. What do you do when you encounter difficulty in understanding speech?
   a. Ask for repetition
   b. Keep quiet
   c. Lip reading
   d. Pretend to have understood everything
   e. Avoid getting into such situation
7. How effortful is it for you to understand speech?
   a. Very much
   b. A little
   c. Not at all
8. What more do you require from your hearing aids?
   a. More audibility in speech
   b. More clarity in speech
   c. Less audibility in speech
   d. More noise cancellation
9. How satisfied are you with the battery life of your hearing aids?
   a. Very satisfied
   b. Satisfied
   c. Neutral
   d. Dissatisfied
   e. Very dissatisfied
10. Do you avoid such listening environment in future?
   a. Yes, definitely
   b. Somewhat
   c. Not at all

What made you to answer the questionnaire? …………………

Thank you very much for answering these questions. Please click on the checkmark to save your answers and exit the survey.

 © 2026. The Author(s).

ಸಂಶೋಧನಾ ಅಧ್ಯಯನ

□□□□ ಎದುರಿಸುತ್ತಿರುವ ಶ್ರವಣ ತೊಂದರೆಗಳನ್ನು ಅರ್ಥಮಾಡಿಕೊಳ್ಳಲು ಈ ಸಮೀಕ್ಷೆಯು ಸಹಾಯಕವಾಗುತ್ತದೆ. ನಿಮ್ಮನ್ನು ಚೆನ್ನಾಗಿ ತಿಳಿದುಕೊಳ್ಳಲು ನಮಗೆ ಸಹಾಯ ಮಾಡಲು ದಯವಿಟ್ಟು 5-10 ನಿಮಿಷಗಳನ್ನು ತೆಗೆದುಕೊಳ್ಳುವುದು

1. ದಯವಿಟ್ಟು ನಿಮ್ಮ ಹೆಸರನ್ನು ಟೈಪ್ ಮಾಡಿ

2. ನೀವು ಪ್ರಸ್ತುತ ಏನು ಮಾಡುತ್ತಿರುವಿರಿ?

a. ಒಬ್ಬರೊಂದಿಗೆ ಸಂಭಾಷಣೆ ಮಾಡುತ್ತಿದ್ದೇನೆ

b. ಅಥವಾ ಹೆಚ್ಚಿನವರೊಂದಿಗೆ ಸಂಭಾಷಣೆ ಮಾಡುತ್ತಿದ್ದೇನೆ

c. ರೆಸ್ಟೋರೆಂಟ್‌ನಲ್ಲಿ ಇರುವುದು ಅಥವಾ ರೆಸ್ಟೋರೆಂಟ್‌ನಲ್ಲಿ ಊಟ ಮಾಡುತ್ತಿದ್ದೇನೆ

d. ಉದ್ಯಾನವನದಲ್ಲಿ ನಡೆದಾಡುತ್ತಿದ್ದೇನೆ

e. ಸಂಗೀತ ಕೇಳುತ್ತಿದ್ದೇನೆ

f. ಸಭೆಯಲ್ಲಿ ಭಾಗವಹಿಸುತ್ತಿದ್ದೇನೆ

g. ಟಿವಿ ನೋಡುತ್ತಿದ್ದೇನೆ

h. ಇತರೆ

3.ನೀವು ಈಗ ಇರುವ ಕೊಠಡಿ ಅಥವಾ ಸ್ಥಳ/ಪರಿಸರವನ್ನು ಹೇಗೆ ವಿವರಿಸುತ್ತೀರಿ?

a. ಸ್ತಬ್ಧ

b. ಸ್ವಲ್ಪ ಗದ್ದಲ

c. ಮಧ್ಯಮ ಗದ್ದಲ

d. ತುಂಬಾ ಗದ್ದಲ

4. ಪ್ರಸ್ತುತ ಸ್ಥಳ/ಪರಿಸರವನ್ನು ನೀವು ಎಷ್ಟು ಬಾರಿ ಎದುರಿಸುತ್ತೀರಿ?

a. ಆಗಾಗ್ಗೆ

b. ಒಂದು ದಿನದಲ್ಲಿ ಅರ್ಧದಷ್ಟು ಸಮಯ

c. ಅಪರೂಪಕ್ಕೆ

5.ಈ ಪರಿಸ್ಥಿತಿಯಲ್ಲಿ ಶ್ರವಣ ಸಾಧನವನ್ನು ಹೊಂದಿಲ್ಲದಿದ್ದರೆ, ನಿಮಗೆ ಹೇಗೆ ಅನಿಸುತ್ತದೆ?

a. ಅಸಹಾಯಕ

b. ನಿರ್ವಹಿಸಲು ಅಸಾಧ್ಯ

c. ಹೇಗಾದರೂ ಸರಿಹೊಂದಿಸಲು ಮತ್ತು ನಿರ್ವಹಿಸಿರಬಹುದು

6.ಪ್ರಸ್ತುತ ಸ್ಥಳ/ಪರಿಸರದಲ್ಲಿ ಶ್ರವಣ ಸಾಧನವು ನಿಮಗೆ ಸಹಾಯ ಮಾಡುತ್ತಿದೆಯೇ?

a. ಸಂಪೂರ್ಣವಾಗಿ

b. ಸ್ವಲ್ಪಮಟ್ಟಿಗೆ ಹೌದು

c. ಇಲ್ಲವೇ ಇಲ್ಲ

□□□□ ಪ್ರಶ್ನೆಗೆ ನಿಮ್ಮ ಉತ್ತರವು ಆಯ್ಕೆ B ಮತ್ತು/ಅಥವಾ C ಆಗಿದ್ದರೆ, ದಯವಿಟ್ಟು ಮುಂದಿನ ಪ್ರಶ್ನೆಗಳನ್ನು ಭರ್ತಿ ಮಾಡಿ

7. ಹಿಯರಿಂಗ್ ಏಡ್ಸ್ / ಶ್ರವಣ ಸಾಧನಗಳನ್ನು ಹಾಕಿಕೊಳ್ಳುವ ಮೂಲಕ ಪ್ರಸ್ತುತ ಪರಿಸ್ಥಿತಿಯನ್ನು ನೀವು ಹೇಗೆ ಭಾವಿಸುತ್ತೀರಿ?

a. ನಾನು ನಿರಾಶೆಗೊಂಡಿದ್ದೇನೆ

b. ನನಗೆ ಮುಜುಗರವಾಯಿತು

c. ನಾನು ಖಿನ್ನತೆಗೆ ಒಳಗಾಗಿದ್ದೇನೆ

9.ನಿಮ್ಮ ಪ್ರಸ್ತುತ ಪರಿಸರದಲ್ಲಿ ಶಬ್ದಗಳ ದಿಕ್ಕನ್ನು ಸ್ಥಳೀಕರಿಸಲು ಅಥವಾ ನಿರ್ಧರಿಸಲು ನಿಮಗೆ ಸಾಧ್ಯವಾಗುತ್ತದೆಯೇ?

a. ಹೌದು, ನಿಖರವಾಗಿ

b. ಸ್ವಲ್ಪಮಟ್ಟಿಗೆ

c. ಇಲ್ಲವೇ ಇಲ್ಲ

10.ನಿಮ್ಮ ಪ್ರಸ್ತುತ ಪರಿಸರದಲ್ಲಿ ಹಿನ್ನೆಲೆ ಶಬ್ದಗಳು ಅಥವಾ ಶಬ್ದಗಳನ್ನು ನೀವು ಕೇಳಲು ಮತ್ತು ಅರ್ಥಮಾಡಿಕೊಳ್ಳಲು ಸಾಧ್ಯವೇ?

a. ಹೌದು, ಸ್ಪಷ್ಟವಾಗಿ

b. ಸ್ವಲ್ಪಮಟ್ಟಿಗೆ

c. ಇಲ್ಲವೇ ಇಲ್ಲ

11.ಇದೀಗ ವ್ಯಕ್ತಿ ಅಥವಾ ಜನರ ಧ್ವನಿ ಎಷ್ಟು ಜೋರಾಗಿ ಧ್ವನಿಸುತ್ತದೆ?

a. ತುಂಬಾ ಜೋರಾಗಿ

b. ಸ್ವಲ್ಪ ಜೋರಾಗಿ

c. ನನಗೆ ಸರಿಯಾಗಿದೆ

d. ಸ್ವಲ್ಪ ಮೃದು

e. ತುಂಬಾ ಮೃದು

12.ಈಗ ಮಾತನಾಡುವುದನ್ನು ನೀವು ಎಷ್ಟು ಚೆನ್ನಾಗಿ ಅರ್ಥಮಾಡಿಕೊಂಡಿದ್ದೀರಿ?

a. ಬಹುತೇಕ ಎಲ್ಲಾ

b. ಏನೋ ಸ್ವಲ್ಪ

c. ಬಹುತೇಕ ಏನೂ ಇಲ್ಲ

13.ಮಾತನಾಡುವುದನ್ನು ಅರ್ಥಮಾಡಿಕೊಳ್ಳಲು ನಿಮಗೆ ಕಷ್ಟವಾದಾಗ ನೀವು ಏನು ಮಾಡುತ್ತೀರಿ?

a. ಪುನರಾವರ್ತನೆಗಾಗಿ ಕೇಳಿ

b. ಮೌನವಾಗಿರಿ

c. ತುಟಿ ನೋಡುವ ಮೂಲಕ ಅರ್ಥಮಾಡಿಕೊಳ್ಳುವುದು

d. ಎಲ್ಲವನ್ನೂ ಅರ್ಥಮಾಡಿಕೊಂಡಂತೆ ನಟಿಸಿ

14.ಮಾತನಾಡುವುದನ್ನು ಅರ್ಥಮಾಡಿಕೊಳ್ಳುವುದು ನಿಮಗೆ ಎಷ್ಟು ಪ್ರಯಾಸದಾಯಕವಾಗಿದೆ?

a. ತುಂಬಾ

b. ಸ್ವಲ್ಪ

c. ಇಲ್ಲವೇ ಇಲ್ಲ

15.ನಿಮ್ಮ ಶ್ರವಣ ಸಾಧನಗಳಿಂದ ನಿಮಗೆ ಇನ್ನೇನು ಬೇಕು?

a. ಮಾತಿನಲ್ಲಿ ಹೆಚ್ಚು ಶ್ರವ್ಯತೆ/ಗಟ್ಟಿತನ/ ಜೋರಾಗಿ

b. ಮಾತಿನಲ್ಲಿ ಹೆಚ್ಚು ಸ್ಪಷ್ಟತೆ

c. ಮಾತಿನಲ್ಲಿ ಕಡಿಮೆ ಶ್ರವ್ಯತೆ/ಗಟ್ಟಿತನ/ ಜೋರಾಗಿ

d. ಹೆಚ್ಚು ಶಬ್ದ ರದ್ದತಿ

16.ನೀವು ದೀರ್ಘಾವಧಿಯವರೆಗೆ ಶ್ರವಣ ಸಾಧನಗಳನ್ನು ಎಷ್ಟು ಆರಾಮದಾಯಕವಾಗಿ ಧರಿಸಿದ್ದೀರಿ?

a. ತುಂಬಾ ಆರಾಮದಾಯಕ

b. ಆರಾಮದಾಯಕ

c. ಸ್ವಲ್ಪ ಅನಾನುಕೂಲ

d. ತುಂಬಾ ಅಹಿತಕರ

17.ಭವಿಷ್ಯದಲ್ಲಿ ನೀವು ಅಂತಹ ಆಲಿಸುವ ವಾತಾವರಣವನ್ನು ತಪ್ಪಿಸುತ್ತೀರಾ?

a. ಹೌದು, ಖಂಡಿತ

b. ಸ್ವಲ್ಪಮಟ್ಟಿಗೆ

c. ಇಲ್ಲವೇ ಇಲ್ಲ
